# Neural Networks Reveal the Impact of the Vibrational Dynamics in the Prediction of the Long-Time Mobility of Molecular Glassformers

**DOI:** 10.3390/ijms23169322

**Published:** 2022-08-18

**Authors:** Antonio Tripodo, Gianfranco Cordella, Francesco Puosi, Marco Malvaldi, Dino Leporini

**Affiliations:** 1 Dipartimento di Fisica “Enrico Fermi”, Università di Pisa, Largo B. Pontecorvo 3, I-56127 Pisa, Italy; 2 Istituto Nazionale di Fisica Nucleare (INFN), Sezione di Pisa, Largo B. Pontecorvo 3, I-56127 Pisa, Italy; 3 Istituto per i Processi Chimico-Fisici-Consiglio Nazionale delle Ricerche (IPCF-CNR), via G. Moruzzi 1, I-56124 Pisa, Italy

**Keywords:** dynamic propensity, glassy system, machine learning, neural network, vibrational dynamics

## Abstract

Two neural networks (NN) are designed to predict the particle mobility of a molecular glassformer in a wide time window ranging from vibrational dynamics to structural relaxation. Both NNs are trained by information concerning the local structure of the environment surrounding a given particle. The only difference in the learning procedure is the inclusion (NN **A**) or not (NN **B**) of the information provided by the fast, vibrational dynamics and quantified by the local Debye–Waller factor. It is found that, for a given temperature, the prediction provided by the NN **A** is more accurate, a finding which is tentatively ascribed to better account of the bond reorientation. Both NNs are found to exhibit impressive and rather comparable performance to predict the four-point susceptibility $\chi_{4}\left(t\right)$ at $\tau_{\alpha }$, a measure of the dynamic heterogeneity of the system.

## 1. Introduction

Upon cooling, if crystallization is avoided, liquids experience a progressive slowing down of their dynamics and finally reach an off-equilibrium, disordered, solid-like state, a glass [1,2]. The glass transition is a matter of intense research to understand the underlying causes.

In particular, the existence of a connection between structure and dynamics is still a matter of debate and up to now, only weak correlations have been reported to support this idea [3,4,5,6,7,8,9,10]. Whether there exists such a connection is still a matter of debate.

Over the last few years, the issue has been investigated by Machine Learning (ML) approaches. ML is a specific subset of artificial intelligence—the science of mimicking human abilities—that instructs a machine how to learn from suitable “training data” and improve from experience, i.e., the size of training data, to make predictions without being explicitly programmed [11]. ML strategies in the field of the glass-forming liquid focus on predicting the dynamical behavior based on local structural features. Starting from the pioneering work by Cubuk et al. [12] relying on support vector machines (SVMs) to predict rearrangement probability in glassy mixtures, more and more cutting edge techniques have been employed to accomplish similar tasks [13,14,15,16,17,18].

When looking for a link between structure and dynamics, it is common practice to resort to the dynamic propensity of a particle, i.e., the displacement of a particle averaged over a suitable ensemble of trajectories starting from a common initial spatial configuration [8,9,10,19,20,21]. The propensity is designed to capture the impact of the structure on the future mobility of a particle. In this context, the ultimate task of clarifying the link between the structure and dynamics translates into predicting the correct behavior of the propensity at long times based on the initial structural information.

To date, the most relevant studies involving the prediction of propensity in glassy systems with ML techniques are, essentially, the following two: The first one was carried out by Bapst et al. [22]. It involves the employment of graph neural networks (GNNs) to predict the propensity in a binary atomic mixture. With a complex and highly advanced ML approach, Bapst et al. were able to outperform previous ML attempts to predict the long-time propensity. The second study was carried out by Boattini et al. [14]. Taking inspiration from the GNN approach, they designed a set of efficient local descriptors thanks to which a much simpler ML architecture such as linear regression is capable of achieving a similar (or even better) performance in predicting the long-time propensity in binary atomic mixtures and hard spheres liquids. However, the quality of the predictions achieved by the GNNs developed by both by Bapst et al. and Boattini et al., even if remarkable, is not perfect and motivates further developments. Most important, the above mentioned studies investigated only *atomic* liquids (single component or mixtures) [13,14,22]. Thus, the performance of the GNNs when tackling connected systems such as *molecular* liquids is unclear. The presence of the molecular strong constraints limiting the particle displacements, e.g., bond lengths and bond-bond angles, is anticipated to be not negligible.

The present paper reports on two distinct advances:

- the test of the approach of Ref. [14] by developing Neural Network (NN) architectures to predict the propensity in a molecular liquid, a melt of fully-flexible short chains. Melts of fully-flexible chains are known to have structural relaxation independent of the chain length [23] and thus are of large interest in polymer science to investigate long-time dynamics with considerable saving of computational effort [24]. Even if the results address conventional linear structural polymers, they are anticipated to provide at least useful hints concerning more complex polymer systems, such as bio-polymers and proteins.
- As further advance, in order to improve the prediction of propensity in glassy systems with ML techniques, we consider as an additional source of information the picosecond vibrational dynamics of the particles wiggling within the transient cage formed by their neighbors (augmented with further information on local structure drawn by Voronoi tessellation and potential energy per particle). Despite the huge difference of time-scales, strong correlations between the vibrational dynamics and the long time structural relaxation, rearranging the cage with considerable spatial distribution of mobility—so-called dynamical heterogeneity (DH)—are well known, see a recent short review [25]. As a matter of fact, the vibrational dynamics inside the cage was one of the first predictors of the long time propensity [19]. We show that this novel scheme improves the accuracy of the prediction, which is found to be significantly temperature-independent.

## 2. Results and Discussion

### 2.1. Structural Relaxation and Propensity

**Figure 1:**
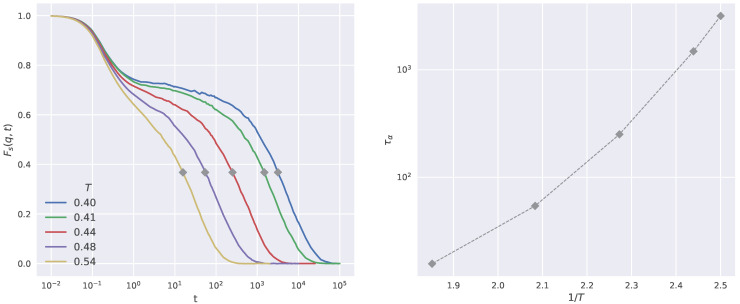
(**Left**): Temperature dependence of ISF. Dots mark the position of the structural relaxation time $\tau_{\alpha }$. (**Right**): Arrhenius plot of the structural relaxation time $\tau_{\alpha }$.

To characterize the dynamics of the system we consider the self part of the intermediate scattering (ISF) function defined as [26]:

(1) $$F_{s}\left(q,t\right)=\frac{1}{N}\langle \sum_{i=1}^{N}e^{-iq\cdot \delta r_{i}\left(t\right)}\rangle$$

where 〈⋯〉 denotes the ensemble average, $\delta r_{i}\left(t\right)$ is the displacement of the *i*-th particle within the time interval *t* and the wave-vector q is chosen so that its modulus corresponds to the first peak of the static structure factor of the system. In an isotropic liquid ISF depends only on the modulus of the wavevector q=||q|| and features the rearrangements of the spatial structure of the fluid over the length scale ∼2π/q. We define $\tau_{\alpha }$, the structural relaxation time, as the time when ISF drops to 1/e, $F_{s}\left(q,t=\tau_{\alpha }\right)=1/e$. The left panel of Figure 1 shows the temperature dependence of the ISF for the investigated system. ISF exhibits the characteristic two-step decay signaling, at intermediate times, the presence of considerable trapping of the monomer in the cage of the surrounding particles. $\tau_{\alpha }$ may be read as the average time to escape from the cage by a monomer. It increases in a non-Arrhenius way by lowering the temperature (Figure 1, right).

**Figure 2:**
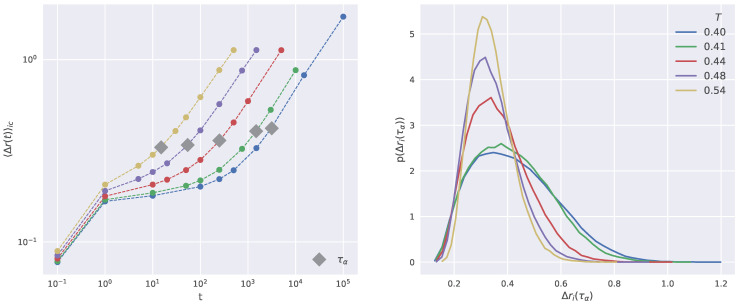
(**Left**) panel: Time-behavior of the particle and ICEs-averaged propensity for all the investigated state points. (**Right**) panel: Probability density function of the particle propensity $\Delta r_{i}\left(\tau_{\alpha }\right)$.

Within ICE it is common practice to characterize the dynamics of the particles in terms of the propensity $\Delta r_{i}\left(t\right)$, the displacement of the *i*-th particle in a time *t* starting from a fixed initial configuration and averaged within a single ICE. Figure 2 plots the temperature dependence of the average propensity ${\langle \Delta r\left(t\right)\rangle }_{ic}$, where the average ${\langle \cdots \rangle }_{ic}$ is taken over all the ICEs and all the particles (left panel), and the probability distribution of the propensity at $t=\tau_{\alpha }$ for all the investigated temperatures (right panel).

### 2.2. Local Descriptors

#### 2.2.1. Structural Descriptors

We minutely described the local environment around a given particle *i* employing a set of handcrafted descriptors based on radial densities and angular functions widely used in ML tasks [14,17,27]. Here, we employ such descriptors in the form of the ones used in Ref. [14].

The radial descriptors consist of weighted particle densities at a distance *r* from the central particle. The radial descriptor of the particle *i* at distance *r* and tolerance δ is defined as [14]:

(2) $$G_{i}^{\left(0\right)}\left(r,\delta \right)=\sum_{j\neq i}e^{-\frac{{\left(r_{ij}-r\right)}^{2}}{2\delta^{2}}}$$

where $r_{ij}$ is the distance between the particle *j* and the central one.

The angular functions take a cue from the bond-orientational order parameters [28] being defined based on a spherical harmonics weighted sum. First, given the central particle *i*, one defines the complex quantity

(3) $$q_{i}\left(r,\delta ,l,m\right)=\frac{1}{Z}\sum_{j\neq i}e^{-\frac{{\left(r_{ij}-r\right)}^{2}}{2\delta^{2}}}Y_{l}^{m}\left(r_{ij}\right)$$

where $Y_{l}^{m}\left(r_{ij}\right)$ are the spherical harmonics of order *l* with −l≤m≤l and *Z* is a normalization factor. Finally, the local angular descriptor is obtained considering a sum over *m* [14]

(4) $$q_{i}^{\left(0\right)}\left(r,\delta ,l\right)=\sqrt{\frac{4\pi }{2l+1}\sum_{m=-l}^{l}{\left|q_{i}\left(r,\delta ,l,m\right)\right|}^{2}}$$

We augmented the local environment description refinement by also considering progressive coarse-grained versions of the descriptors. Being $x_{i}^{\left(0\right)}$ a non-coarse-grained radial or angular local descriptors of particle *i*, we define the *n*-th generation coarse-grained version of it as

(5) $$x_{i}^{\left(n\right)}=\frac{\sum_{j:r_{ij}<r_{c}}x_{j}^{\left(n-1\right)}e^{-r_{ij}/r_{c}}}{\sum_{j:r_{ij}<r_{c}}e^{-r_{ij}/r_{c}}}$$

where $r_{c}$ is the radius of coarse-graining. Such coarse-graining was introduced by Boattini et al. [14], inspired by the pioneering result obtained with Graph Neural Network [22], and allows to probe the local structure at different length-scales.

In total, we employed 292 0-th order structural descriptors: 100 radial and 192 angular. Radial descriptors where measured in 60 equally spaced spherical shells of width δ=0.025 in the interval r∈[0.5,2], 20 shells of width δ=0.05 in the interval r∈(2,3] and 20 shells of width δ=0.1 in the interval r∈(3,5]. Angular descriptors were measured in 16 equally spaced spherical shells of width δ=0.1 in the interval r∈[0.5,2.5] with l=1,2…12.

In this work, we employ two generations of coarse-graining, i.e., n=1,2 in Equation (5), and a radius $r_{c}=2.5$, thus reaching a total of 876 structural descriptors. Changing the value of the coarse-graining radius does not affect significantly the results [14].

In addition to the above radial and angular sets, we enrich the description of the local environment by considering: (i) the volume, surface and asphericity of the Voronoi polyhedron associated to each particle, (ii) the per-particle total potential energy evaluated by summing all the pair contributions for each particle.

#### 2.2.2. Short-Time Dynamics Descriptor

Rather than adding more and more structural descriptors, we consider the possibility of enriching the information on a single particle with its short-time mobility. To this aim, we employ $\Delta r_{i}^{2}\left(t=1\right)$, the squared displacement of the *i*-th particle in a time t=1 starting from a fixed initial configuration and averaged within a single ICE. Note that t=1 corresponds to a few picoseconds [29]. We refer to this quantity as the local Debye–Waller (DW) factor for the i-th particle.

It has been shown that this quantity can predict the correct spatial distribution of the long time propensity if one considers particles whose local DW exceeds a certain threshold, reminiscent of a Lindemann-like criterion [19,30,31] However, in general, the local DW alone does not provide fully satisfactory predictions of the long time behavior of the propensity of a particle, as recently demonstrated [22].

### 2.3. Neural Network

NNs are a computing architecture, designed to mimic the behavior of the human brain [11,32]. Their fundamental units, namely the nodes, work as biological neurons. They are arranged in hierarchical structures named “layers”. Layers are stacked together to form a network. The first and the last layers are respectively called input and output layer while all the layers standing between them are called hidden layers. Each node linearly combines all the information coming from the nodes belonging to the previous layer and passes it to the next layer via a non-linear “activation” function. In our case, we chose a Rectified Linear (ReLu) [11,32] activation function for all the nodes in the nets.

In this work, we employed two NNs to carry out the propensity prediction, namely **A** and **B**. **A** and **B** differ only for their input: NN **A** takes in input all the structural information given by all the descriptors discussed in Section 2.2.1 plus the information on the short time dynamics provided by the local DW factor while NN **B** keeps track only of the structural information, i.e., without the local DW.

For each temperature, depending on the system relaxation time, a set of 10 time intervals has been chosen ranging from the ballistic regime t≈0.01 up to the early diffusive regime $t\gg \tau_{\alpha }$ (see left panel of Figure 2). To this stage, a remark concerning the role of time flow in NNs is in order. Indeed, NN manages the information related to two distinct times, say $t_{1}$ and $t_{2}$, on an equal footing with no consideration of whether $t_{1}<t_{2}$ or vice versa. Past and future are meaningless concepts in the NN. On the other hand, we refrain from comparing the performances of NN **A** and NN **B** for times shorter than t=1, where the local DW is evaluated. The latter is built up by the series of particle displacements occurring in the time window between the initial time and t=1 and then an increasingly better performance of NN **A** on approaching t=1 from shorter times is anticipated.

For every particle *i*, the propensity is computed for this set of time interval and the resulting vector is the target for both the neural networks. This means that the prediction of the propensity at all the investigated times happens simultaneously. This approach is different from the one adopted in previous works where the prediction is made one time interval at a time [13,14,22]. Such a choice has been made to force the network to produce a model that works at all the time-scales.

Both networks **A** and **B** consist of three hidden layers, in addition to the input and output layers. Each hidden layer is composed of 16 nodes. To control the overfitting, we employed in both networks a drop-out layer between the second and third hidden layer, and make use of a $L_{2}$ regularization of the layers weights [11,32].

Before going through the NN, each parameter $x_{i}$ is standardized by computing

(6) $$x_{i}^{\mathrm{st}.}=\frac{x_{i}-\bar{x}}{\sigma_{x}}$$

where $\bar{x}$ and $\sigma_{x}$ are respectively the average and the standard deviation of the considered parameter. This transformation ensures an a priori equal weight to all the parameters in the training procedure [11,32]. After the standardization, for each temperature we have a data set consisting of rows of all the local descriptors for all the particles and all the independent runs. Thus, each data set is randomly row-wise divided in a training set and a test set with a 80:20 ratio. The training set is devoted only to the training of the NNs while the test set is employed to test its performance.

We train the NNs using the Python package TensorFlow [33] and employed the Adam optimizer [34] for the minimization of the mean squared error loss function.

### 2.4. Predicting the Propensity

**Figure 3:**
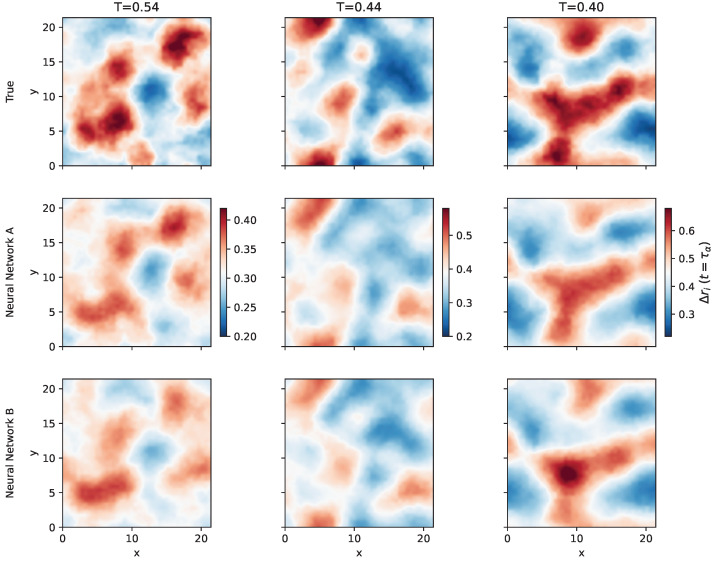
Comparison of the true and the predicted propensity maps of the neural networks **A** and **B** for three different temperatures. Both NNs are trained at a given temperature and the predictions inspected at the *same* temperature. Maps are obtained by interpolating the propensities of particles belonging to a slice of height h=1 along the *z* axis of the simulation cubic box. The predicted propensity are obtained with (second row) and without (third row) the local DW in the input data set. Colorbars refer to the entire columns (i.e., the given temperature).

Here we examine the performance of the NNs in the prediction of the propensity at various timescales. First, we train the NN at a given temperature and inspect the prediction at the *same* temperature. Figure 3 shows a visual comparison of the true and the predicted values of the propensity at $t=\tau_{\alpha }$ in a two dimensional slice of the sample at three distinct temperatures. The two NNs can predict with significant accuracy the propensity at the structural relaxation timescale. It is also worth noting that the two NNs reproduce nicely the spatial correlation of propensity, in particular the soft spots of the system where the most mobile particles reside.

Figure 3 is encouraging but it misses any statistical significance. To provide it, we consider the Pearson correlation coefficient ρ(t) between the true and predicted propensity at time *t*.

It is defined as

(7) $$\rho \left(t\right)=\frac{\mathrm{Cov}(v(t),w(t))}{\sigma_{v(t)}\sigma_{w(t)}}$$

where v(t) is the true propensity list at a given time *t* and *w* is the predicted one at the same time. Cov(v(t),w(t)) is the covariance between v(t) and w(t), while $\sigma_{v(t)}$ and $\sigma_{w(t)}$ are the two standard deviations. The covariance and the standard deviations are computed considering all the true and predicted propensities corresponding to the test set.

The NN exact prediction of the propensity yields ρ=1, whereas the random assignment leads to ρ=0.

**Figure 4:**
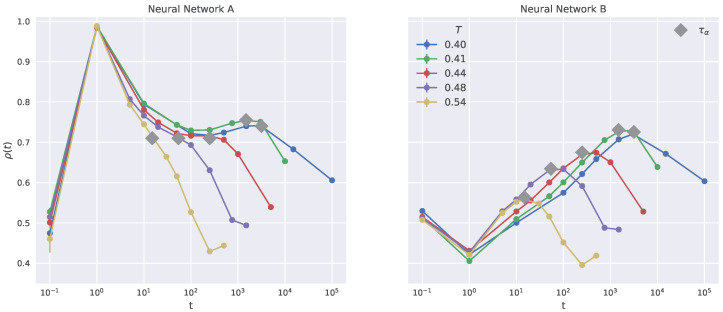
Pearson correlation coefficient of the propensity prediction for all the investigated state point with Neural Network **A** (**left** panel) and **B** (**right** panel). Gray diamonds mark the position of the structural relaxation time of the given state point.

Figure 4 plots the time behavior of the Pearson correlation coefficient of the two investigated NNs. First, let us focus on the NN **B**, i.e., the one considering only the structural descriptors discussed in Section 2.2.1 and not resorting to information about the local DW (right panel). The correlation agrees with the reported literature and exhibits a well-defined peak at $∼\tau_{\alpha }$ [13,14,22]. In particular, at very short times (t∼0.1) the correlation is rather small and even slightly less than the one in hard-sphere systems [13]. At intermediate times, at the onset of the cage regime (t≈1), corresponding to the start of the quasi-plateau region observed in the left panel of Figure 2, we observe a minimum of the Pearson correlation. Such a minimum is also found in the hard spheres system [13]. Only when the central particle starts to notch the surrounding cage, it is possible to observe a more distinct connection of the propensity with the local structure. In fact, at later times, for t>1, the correlation grows and reaches its maximum at approximately $t∼\tau_{\alpha }$ for all the temperatures. We note that the maximum of the correlation, and therefore the goodness of the propensity prediction, is strongly dependent on the temperature: at lower temperatures we observe higher correlation. This was attributed to a reduction of thermal noise [22] perturbing the target propensity or the structural descriptors. Alternatively, it cannot be ruled out that the role of the local structure becomes more and more relevant on decreasing the temperature, as reported in several works [3,4,5,6,7,8,9,10]. After the structural relaxation time ($t>\tau_{\alpha }$), correlations from the initial structure vanish and the decay of the prediction performance is observed.

The performance of the NN **A** is presented in Figure 4 (left). We remind that NN **A** considers the local DW as an additional environmental descriptor. One notices that for a given temperature the correlation is higher than in the case of NN **B**, in a wide time window up to the structural relaxation time $\tau_{\alpha }$.

Going to a more detailed inspection, one sees that a peak of correlation is found at t=1. The latter is because the particle local DW and the squared propensity at t=1 are perfectly correlated so it is natural for the NN to give an high relative weight to it. Furthermore, the $L_{2}$ regularization of the layers weights, see Section 2.3, forces the NN to predict with as few parameters as possible, leading to the major role of the DW factor in the cage regime. For t>10, one sees that the correlation growth provided by NN **B** up to $\tau_{\alpha }$ is now replaced by a nearly temperature-independent plateau. For $t≳\tau_{\alpha }$, the performance by NN **A** decays similarly to the one by NN **B**.

**Figure 5:**
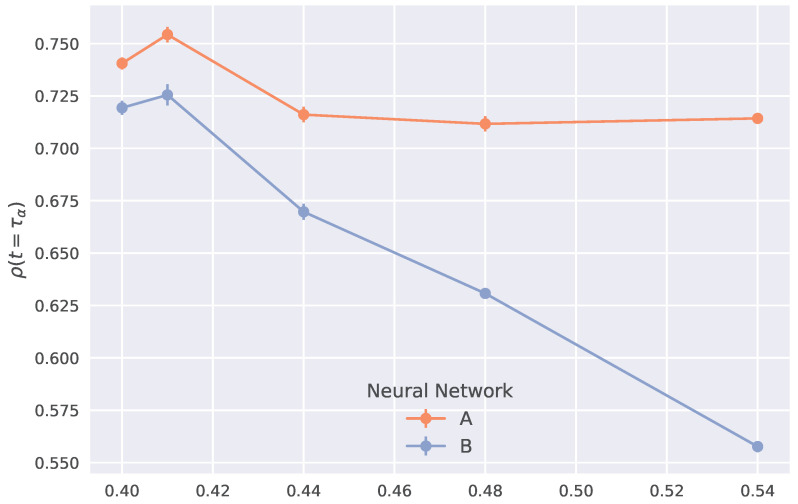
Comparison of the prediction accuracy at $t=\tau_{\alpha }$ in either the presence (NN **A**) or the absence (NN **B**) of the local DW in the input features data-set.

Since we are interested in the long-time propensity, to better illustrate the advantage of including the local DW in the local descriptors, we focus on the accuracy of the predictions provided by the two NNs at the structural relaxation time. To this aim, Figure 5 compares the correlation coefficient of NN **A** and **B** at different temperatures. It is seen that NN **A** provides a nearly temperature-independent correlation, higher than the one of NN **B**, even at temperatures rather close to $T_{g}$ ($T_{g}\approx 0.395$ [35]). More explicitly:

- the sole consideration of the particle position at a given time provides poor account of long-time large particle displacement in connected systems, especially at high temperatures;
- the information encoded in the local DW counteracts the drawback.

As a tentative explanation, we speculate that gathering even accurate structural information at a given time does not lead to proper account of the long-time bond reorientation which requires, even in the present simple molecular model, some degree of cooperativity between bonded atoms. The local DW, which is certainly affected by the molecular connectivity, seems able to compensate the limitations of the structural information.

### 2.5. Dynamical Heterogeneity

In order to provide better statistical basis to the DH apparent in Figure 3, we now assess the NN performance to predict the spatial correlation of the propensity by computing the four-point susceptibility $\chi_{4}\left(t\right)$ of the propensity which is defined as [36,37]:

(8) $$\chi_{4}\left(t\right)=\frac{1}{N}\left[\sum_{i,j=1}^{N}{\langle q_{i}^{a}q_{j}^{a}\rangle }_{ic}-{\left(\sum_{i=1}^{N}{\langle q_{i}^{a}\rangle }_{ic}\right)}^{2}\right]$$

where $q_{i}^{a}=1$ if $\Delta r_{i}\left(t\right)>a$ and 0 otherwise, and *a* is chosen as the mean displacement modulus at $t=\tau_{\alpha }$ (for instance a=0.4286 for T=0.40). The $\chi_{4}\left(t\right)$ gives a measure of the correlated motion size [37] and then offers a DH measure [38]. This peculiar choice of a temperature dependent probe-length *a* has been made to focus on DH at the $\tau_{\alpha }$ time scale. Different choices of *a* were considered including temperature independent values a=0.4,0.5,0.6 designed to investigate the DH at a fixed legth-scale. It has been observed that, even if the $\chi_{4}$ time behavior is modified, the quality of the NNs prediction remains unchanged.

**Figure 6:**
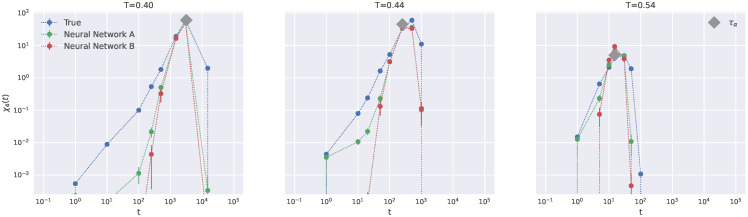
Comparison of the true four-point correlation function $\chi_{4}\left(t\right)$ with the predictions provided by the NNs **A** and **B** at different temperatures.

Figure 6 presents the comparison between the four-point susceptibility computed with the true propensity and the predictions achieved via the NNs **A** and **B** at different temperatures. We note that, as expected, the local DW factor slightly improves the accuracy of the predicted $\chi_{4}\left(t\right)$ at intermediate time scales (t≳1). More remarkably, it is seen that both NNs are rather accurate at $t=\tau_{\alpha }$, corresponding approximately to the maximum of $\chi_{4}\left(t\right)$. This provides sound statistical basis for the conclusions which are hinted at by comparing the true and the predicted DH shown in Figure 3.

The prediction of $\chi_{4}\left(\tau_{\alpha }\right)$ in a binary atomic mixture provided by GNN has been reported as less effective [22].

### 2.6. Generalization to Other Temperatures

How effective is the NN prediction of the propensity at a temperature which is *different* from the one at which training has occurred?

**Figure 7:**
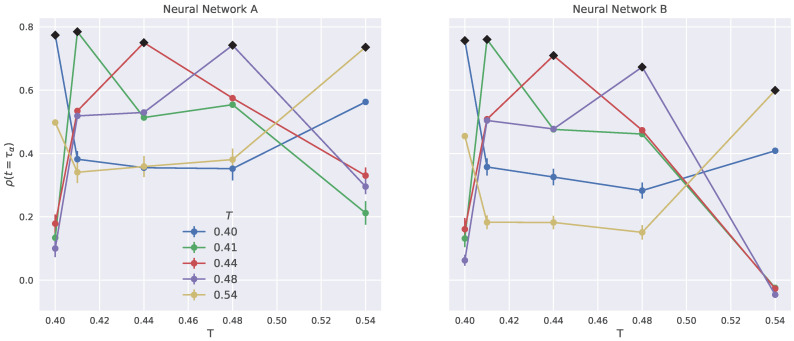
Performance of the NN prediction at $t=\tau_{\alpha }$ for temperatures being *different* from the training one. The training temperature is marked with black diamonds on the curves.

Figure 7 addresses this question for the propensity at $t=\tau_{\alpha }$ for NNs **A** and **B**. For both NNs, given a temperature range of interest, there is some indication suggesting to train the NNs at intermediate temperatures. This agrees with other studies carried out in a binary atomic mixture [22]. We find no clear indications that NN **A** provides better predictions than NN **B**.

## 3. Methods and Materials

We performed molecular dynamics (MD) numerical simulations of a three-dimensional polymer melt of fully-flexible, i.e., with no bond-bond bending or torsional potentials, linear pentamers with a total number of monomers N= 10,000. Non-bonded monomers interact via a Lennard–Jones potential [26] while bonded monomers interact via an harmonic potential $U^{b}\left(r\right)=k{\left(r-r_{0}\right)}^{2}$ where $r_{0}=0.97\sigma$ is the rest length and the stiffness *k* is set to $555.5\epsilon /\sigma^{2}$ with σ being the location of the 0 of the Lennard–Jones potential and ϵ the depth of its minimum. The Lennard–Jones potential is truncated at r=2.5σ for computational convenience. It is also shifted to grant continuity at r=2.5σ. All simulations were carried out with the open-source software LAMMPS [39,40]. From here on, throughout the text we employ dimensionless units based on the parameters of the Lennard–Jones potential.

We equilibrated the system at a constant number density ρ=1.02 for 5 different temperatures approaching the glass transition of the model ($T_{g}\approx 0.395$ [35]). For each state point we considered 10 independent runs to achieve statistical significance. Different runs have the same constant number of particles *N*, volume *V* and temperature *T*. The equilibration runs lasted not less than $3\tau_{ee}$, where $\tau_{ee}$ is the end to end vector autocorrelation function decay time [41].

After equilibration, production runs were carried out at each state point with constant number of particles N, volume V and energy *E*. In parallel, for each of the investigated temperatures and for every independent run, we generated an isoconfigurational ensemble (ICE) consisting of 40 trajectories starting from a common initial spatial configuration of the particles. The trajectories differ one from another by the starting particles velocities which are randomly chosen from the Maxwell–Boltzmann distribution at the considered temperature. ICE allows emphasizing the role of the initial structure of a liquid in influencing future dynamics.

## 4. Conclusions

The present study reports on the characterization of two NNs designed to predict the propensity of a molecular glassforming system in a wide time window ranging from vibrational dynamics to structural relaxation. Both NNs consider information drawn from the local structure of the environment surrounding a given particle. The only difference is the inclusion (NN **A**) or not (NN **B**) of the information provided by the fast, vibrational dynamics and quantified by the local DW. The main result is that, for a given temperature, the prediction of the propensity provided by the NN **A** is more accurate in a wide time window between the nearly-ballistic regime (t∼0.1) and the structural relaxation time $\tau_{\alpha }$. In particular, when the predictions of the propensities at the structural relaxation time of the two NNs are compared, it is found that the accuracy of NN **A** is higher and almost temperature-independent. We speculate that gathering even accurate structural information at a given time does not lead to proper account of the long-time bond reorientation which requires cooperativity. It appears that the local DW is able to compensate the limitations of the structural information taking naturally into account for the molecular connectivity.

Our study demonstrates that ML algorithms designed to predict the long-term dynamics of a molecular glass-forming system based on the structural information, benefit from the addiction of the fast dynamics information. It will be of interest to study its impact in much more complex ML architectures such as convolutional NNs or a GNNs.

## Data Availability

The data presented in this study are available on request from the corresponding author.

## Abbreviations

The following abbreviations are used in this manuscript:

DH	Dynamical Heterogeneity
DW	Debye–Waller
GNN	Graph Neural Network
ICE	Isoconfigurational Ensemble
ISF	Intermediate Scattering Function
ML	Machine Learning
NN	Neural Network
